# Purpose‐in‐life associates with later onset and lower hazard for cognitive impairment in a population‐based, longitudinal cohort of diverse participants

**DOI:** 10.1002/alz.088289

**Published:** 2025-01-03

**Authors:** Nicholas C Howard, Ekaterina S Gerasimov, Thomas S. Wingo, Aliza P. Wingo

**Affiliations:** ^1^ Emory University, Atlanta, GA USA; ^2^ Emory University School of Medicine, Atlanta, GA USA; ^3^ Department of Human Genetics, Emory University School of Medicine, Atlanta, GA USA

## Abstract

**Background:**

Purpose‐in‐life (PiL) refers to the tendency to derive purpose and meaning in life. Higher PiL was associated with lower risk for developing Alzheimer’s dementia in older white participants (age 70s‐80s). It is unclear, however, whether the protective effect of PiL can be observed as early as in the 50s and 60s, and whether PiL is associated with a delay in onset age of cognitive impairment. Here, we investigated these questions in the population‐based cohort of diverse participants recruited by the Health and Retirement Study.

**Method:**

13,689 participants (10,114 White, 1837 Black, 1335 Hispanic, 405 Other) with normal cognitive status at baseline, PiL assessment, longitudinal depression and cognitive test scores, and genetic data were included. Cognitive performance was assessed biennially with the modified Telephone Interview for Cognitive Status (mTICS) over 15 follow‐up years. The mTICS includes immediate and delay recall, working memory, attention, and processing speed. Cognitive impairment was defined as having mTICS<12, which is a psychometrically validated threshold. Ethnicity‐stratified Cox proportional hazards was performed to assess the association between baseline PiL and incident of cognitive impairment. Linear regressions were performed to examine the association between PiL and onset age of cognitive impairment. APOE e4 allele was additionally adjusted for in the above regression models.

**Result:**

Within each of the ethnicity groups, higher PiL was associated with lower hazard for developing cognitive impairment after adjusting for sex, age at baseline, education, and longitudinal depression (HR = 0.86, p<0.001). This association remained significant after we adjusted for APOE e4 in addition to the above covariates. APOE e4 was not associated with PiL. Furthermore, higher PiL was associated with a later onset age of cognitive impairment after adjusting for sex, age at baseline, education, and longitudinal depression (b = 0.19, p = 0.02).

**Conclusion:**

Higher PiL was associated with lower risk for developing cognitive impairment in diverse populations. Furthermore, this is the first study to show that higher PiL was associated with a later onset age of cognitive impairment in different ethnicities. Interventions that foster PiL can potentially reduce the risk of cognitive impairment and delay its onset age.

